# Phylogenetic Perspectives and Ethnobotanical Insights on Wild Edible Plants of the Mediterranean, Middle East, and North Africa

**DOI:** 10.3390/foods14030465

**Published:** 2025-02-01

**Authors:** Mousaab Alrhmoun, Naji Sulaiman, Andrea Pieroni

**Affiliations:** 1 University of Gastronomic Sciences, 12042 Pollenzo, Italy; a.pieroni@unisg.it; 2 Faculty of Agricultural, Environmental and Food Sciences, Free University of Bolzano, 39100 Bolzano, Italy; 3 Department of Medical Analysis, Tishk International University, Erbil 4001, Iraq

**Keywords:** biogeographical zones, cultural exchanges, ethnobotany, Levant, Mediterranean, phylogenetic relationships, wild plant diversity

## Abstract

This study investigates the phylogenetic and geographical distribution of wild food plants (WFPs) across 30 Mediterranean and North African (MENA) regions, focusing on the intersection of evolutionary lineage, ecological adaptation, and cultural utilization. A phylogenetic analysis of 111 genera of WFPs used in traditional diets reveals clusters reflecting shared ancestry, functional adaptations, and ecological resilience. Key regions such as Lebanon and Ikaria stand out as potential centers for the diversity of wild food plant use, suggesting that the Eastern Mediterranean may be a primary origin area, especially for species adapted to semi-arid climates. Major plant families including *Lamiaceae*, *Rosaceae*, and *Fabaceae* form distinct clusters that underscore their common ancestry and adaptability, making them foundational to traditional diets and medicinal applications across various environments. Geographical analysis indicates historical connections, such as those between Malta and Egypt, supporting the hypothesis that ancient trade routes influenced the spread and cultural exchange of wild food plant use across the Mediterranean. The study emphasizes the integration of phylogenetic and ethnobotanical perspectives, shedding light on how biodiversity, ecological adaptation, and cultural practices intersect in these regions. This research demonstrates that WFPs serve as both ecological and cultural assets, crucial for preserving traditional diets and supporting biodiversity conservation amid environmental changes. Integrating evolutionary and cultural knowledge can enrich ecological understanding and contribute to the sustainable use of plant resources in the MENA regions.

## 1. Introduction

The Mediterranean basin, recognized as one of the world’s 35 biodiversity hotspots, is home to approximately 25,000 species of vascular plants, with nearly half endemic to the region [1,2]. This rich floral diversity is critical not only for maintaining ecological balance but also for supporting human well-being through the provision of vital resources, including food, medicine, and ecosystem services [3]. Given the ecological significance of wild food plants, particularly in the context of changing environmental conditions, there is a pressing need for comprehensive studies that evaluate their distribution, ecological roles, and potential for sustainable use.

Wild plants have served as a food source for people in the Mediterranean since ancient times [4]. Archaeobotanical studies demonstrate that wild plant foraging occurred in Southern Syria as early as 10,000 B.C. during the Early Pre-Pottery Neolithic age, where species such as *Malva* spp. were gathered [5]. The foraging activity has moved with people migrating from one region to another across the Mediterranean, leading to a marriage between various local foraging cultures [6]. The wild plant-associated local ecological knowledge has noticeably decreased globally in the past decades, particularly along the Mediterranean and the Middle East, despite its crucial role during periods of food insecurity [7,8,9]. However, recent years have witnessed a resurgence in the use of wild plants, not only among local communities but also across other sectors, including restaurants and ecotourism [10,11].

The MENA regions exhibit significant diversity in ecological and cultural landscapes, leading to varying conservation approaches for wild plant species. In the Mediterranean, the historical interplay between agriculture, local economies, and cultural practices has shaped the flora, with many WFPs being deeply intertwined with the traditional diets, medicinal practices, and livelihoods of various ethnic groups [1,3,12]. In contrast, North African regions are facing increasing challenges from urbanization, agricultural expansion, and climate change, which threaten traditional practices and wild plant populations. The interplay of economic development and cultural heritage has profound implications for the conservation of WFPs [12,13,14]. Changes in socio-economic conditions can lead to either increased protection of plant diversity or intensified exploitation [15,16]. Understanding this historical and ethnobotanical context is essential for developing effective conservation strategies tailored to the specific challenges faced by wild plant species in these regions.

Furthermore, the threats to WFPs differ significantly across the MENA, necessitating a nuanced understanding of these variances to inform conservation efforts [2,15,17,18]. Factors such as habitat loss, soil degradation, invasive species, overharvesting, and climate change impact WFPs in distinctive ways [1,3,19]. Thus, the evaluation of these threats must take into account local ecological and socio-economic contexts. By examining how WFPs in these regions have been historically utilized and prioritized for conservation, effective approaches can be identified to safeguard their biodiversity and promote sustainable use, ensuring that these valuable resources are preserved for future generations.

The distribution of wild plant species across the Mediterranean and North African regions is subject to a complex interplay of ecological, socio-demographic, and technological factors [20,21]. Ecologically, changes in climate, particularly rising temperatures, altered precipitation patterns, and the increased frequency of extreme weather events has caused shifts in habitat suitability for many wild plant species [3,22]. These changes force species to adapt, migrate, or face local extinction. Species with specific habitat requirements, such as those adapted to cooler mountainous environments, are particularly vulnerable to warming temperatures, while others, such as those suited to arid conditions, may experience range expansions [1,23].

In addition to climate, ecological processes like soil erosion, overgrazing, and the spread of invasive species further exacerbate the challenges faced by native wild plant communities [24]. Invasive species, often introduced through human activity, outcompete native flora, leading to significant shifts in plant community composition [23,25]. Families like *Asteraceae*, *Lamiaceae*, and *Poaceae*, which are often more resilient to environmental fluctuations, tend to dominate in such disturbed ecosystems, while more specialized or less adaptable plant families may decline [26].

Socio-demographic changes, including rapid urbanization, agricultural expansion, and shifts in land-use practices, also play a crucial role in shaping wild plant distribution [27,28]. Traditional land management practices, historically centered around sustainable agriculture and the conservation of natural resources, are increasingly being replaced by intensive farming, urban sprawl, and the over-exploitation of plant resources [20]. Consequently, wild plant populations have significantly diminished in regions undergoing substantial human pressure.

Traditional ecological knowledge (TEK), once a cornerstone of sustainable wild plant use, is also in decline [29,30]. This knowledge passed down through generations, guided local communities in sustainably harvesting WFPs for food, medicine, and other uses. However, as younger generations migrate to urban centers and adopt modern lifestyles, the connection between cultural heritage and biodiversity conservation weakens. The loss of this knowledge threatens the sustainable use of wild plant resources and undermines efforts to develop conservation strategies that integrate traditional practices with modern ecological science [31,32]. Protecting TEK is therefore critical, not only for conservation but also for maintaining cultural diversity.

This study aims to develop a comprehensive dataset encompassing 30 sites across the MENA regions, focusing on the top frequently cited WFPs at each location. Through a scientific evaluation of these WFPs using phylogenetic analyses and biogeographic patterns, the study seeks to provide new insights into their evolutionary history and ecological significance. By examining the relationships between these plants, their habitats, and the cultural practices surrounding their use, we aim to identify key drivers of plant distribution and resilience. This approach will not only deepen our understanding of wild plant diversity but also inform targeted conservation strategies, particularly in regions where biodiversity is threatened by socio-economic and environmental pressures.

By integrating ethnobotanical knowledge with modern ecological research, this study will contribute to the development of sustainable management practices that respect local traditions while promoting biodiversity conservation. Our findings will provide valuable insights for policymakers, conservationists, and local communities, helping to ensure the preservation of these vital resources for future generations.

## 2. Materials and Methods

### 2.1. Selection and Documentation of WFPs in Ethnobotanical Practices

**Figure 1:**
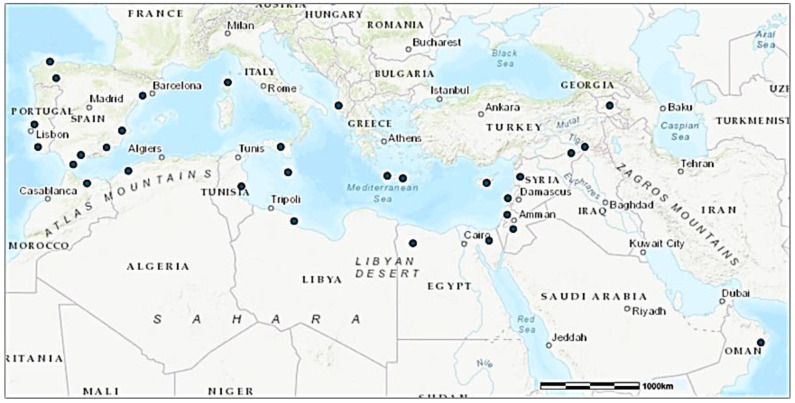
The MENA (Mediterranean and North Africa) field study areas used in the comparative analysis (Central Ikaria (IKA), Central Crete (CRE), Lebanon (LEB), Tartus (SYR), Sidi Bouzid (TUN), Sicily (SIC), Central Armenia (ARM), Northern West Bank (PAL), Syrian–Turkish borderland (ASS), Kurdistan region of Iraq (KUR), Andalusia (ANSPA), Sanabria (SANSPA), Caurel (CAUSPA), Pilona (PILSPA), Picos de Europa (PDESPA), Campoo (CAMSPA), Montesinho (MONPOR), Catalonia (CASPA), Corsica (FRA), Alentejo (ALEPOR), Cyprus (CYP), Malta (MAL), Rif Mountains (MOR), Nile Delta (NDEGY), Siwa Oasis (SOEGY), Jordan Valley (JOR), Cyrenaica (LIB), Albanian South-East (ALB), Oman Mountains (OMA), and Western Algeria (ALG)).

In this study, we compiled a comprehensive list of 111 WFPs utilized in the ethnobotanical practices of diverse populations across the MENA regions (Figure 1). The selection process involved an extensive review of the existing literature, including ethnobotanical studies, traditional ecological knowledge, and field surveys conducted with local communities. We considered all recorded/used WFP genera in these sources, ensuring that the selected species not only represent significant cultural practices but also have documented uses in food and medicine.

To check the widespread edible use of the WFPs in the study area, besides our own studies database that included information from nearly more than 77 ethnobotanical sources, more than 40 additional ethnobotanical works from study countries have been consulted, and a database has been created covering information about 21 Mediterranean and North African countries with both databases. Final data about 111 plant species has been included in the analysis presented in the paper.

Table 1 provides detailed information about the sites included in this research, outlining the countries, main ethnic groups, predominant religions, and languages spoken by the populations in the MENA regions. These data were sourced from a variety of reputable ethnobotanical studies and floristic databases, which ensured the reliability and relevance of the plant data.

Based on the references presented in Table 1 and the database linked to each country, we compiled a list of botanical families associated with the selected WFPs and developed a matrix table. This matrix categorizes the plants according to their respective families and highlights their primary uses in food. Furthermore, Table 2 provides a detailed overview of the botanical genera, including information on the specific uses of these plants in food preparation, their cultural significance, and their frequency of occurrence across the various study sites.

### 2.2. Statistical Analysis

Descriptive statistics were conducted using SAS 9.4 (SAS Institute Inc., Cary, NC, USA), employing PROC FREQ to generate frequency distributions for the occurrence of WFPs across the study sites located in eight regions of the Mediterranean. This analysis provided insights into the prevalence and distribution patterns of the wild plant species, allowing for an initial understanding of plant diversity across the studied regions. Additionally, chi-square tests were performed to examine the relationships between categorical variables, assessing significant associations in wild plant occurrences among different regions.

Following the descriptive analysis, cluster analysis was conducted using R. version (4.4.2) to explore the relationships among the study sites based on their wild plant compositions. Hierarchical clustering was performed using the (hclust function), with a distance matrix computed based on Euclidean distance. This analysis identified distinct groupings among the sites, which were visualized in a dendrogram to illustrate the clustering structure. The optimal number of clusters was determined using the elbow method, allowing for the selection of an appropriate clustering solution.

The final data matrix was converted into Nexus format using MESQUITE Version (3.7), facilitating further phylogenetic analyses. A heuristic add-and-rearrange method was initially employed, using the subtree pruning and regrafting (SPR) rearranger with a maximum of 10,000 equally good trees. Bayesian analysis was performed in MrBayes 3.2.6 with Markov chain Monte Carlo (MCMC) analysis conducted for 50,000,000 generations to assess convergence and stationarity. The output was subsequently verified using Tracer 1.7.1. A maximum likelihood (ML) phylogenetic tree was constructed using the IQ-TREE web server (http://iqtree.cibiv.univie.ac.at, accessed 10 October 2024), incorporating 10,000 ultrafast bootstrap replicates to evaluate the robustness of the tree topology.

## 3. Results

### 3.1. Distribution of Wild Food Plants Across the Study Site

**Figure 2:**
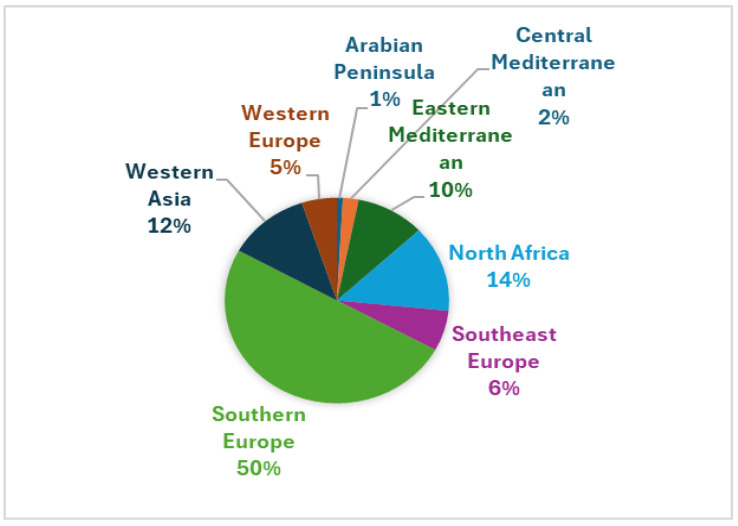
Geographic distribution of WFPs in the MENA regions documented in the study area. In Southern Europe, we identified Sicily (SIC), Andalusia (ANSPA), Sanabria (SANSPA), Caurel (CAUSPA), Pilona (PILSPA), Picos de Europa (PDESPA), Campoo (CAMSPA), Montesinho (MONPOR), Catalonia (CASPA), and Corsica (FRA). Western Europe is represented by Alentejo (ALEPOR). The Eastern Mediterranean includes Central Ikaria (IKA), Central Crete (CRE), and Cyprus (CYP), while Southeast Europe covers Central Armenia (ARM) and the Albanian South-East (ALB). In the Central Mediterranean, Malta (MAL) was identified as a key site. North Africa comprises Sidi Bouzid (TUN), the Rif Mountains (MOR), Western Algeria (ALG), Cyrenaica (LIB), the Nile Delta (NDEGY), and the Siwa Oasis (SOEGY). The Western Asia region includes Lebanon (LEB), Tartus (SYR), the Syrian–Turkish borderland (ASS), the Jordan Valley (JOR), and the Kurdistan region (KUR). Finally, the Arabian Peninsula is represented by the Oman Mountains (OMA).

Our study identified 111 WFPs using data from the reviewed literature across the MENA regions (Figure 1). The distribution varied significantly by region, reflecting the area’s ecological and cultural diversity. Southern Europe, encompassing sites such as Sicily (SIC), Andalusia (ANSPA), and Sanabria (SANSPA), had the highest representation, comprising nearly 49% of the total citations. North Africa, with locations like Tunisia (TUN), Morocco (MOR), and Algeria (ALG), contributed around 13%. Western Asia, including regions like Lebanon (LEB), Syria (SYR), and Jordan (JOR), accounted for approximately 12%. The East-Central Mediterranean, represented by sites like Ikaria (IKA) and Crete (CRE), contributed 10%, indicating consistent use of WFPs in the area. Southeast Europe and Western Europe made up 6% and 5%, respectively, with moderate usage observed in sites such as the Albanian Riviera (ALB) and Alentejo (ALEPOR). The Central Mediterranean, represented by Malta (MAL), accounted for 2%, while the Arabian Peninsula showed the lowest representation at less than 1% (Figure 2).

**Figure 3:**
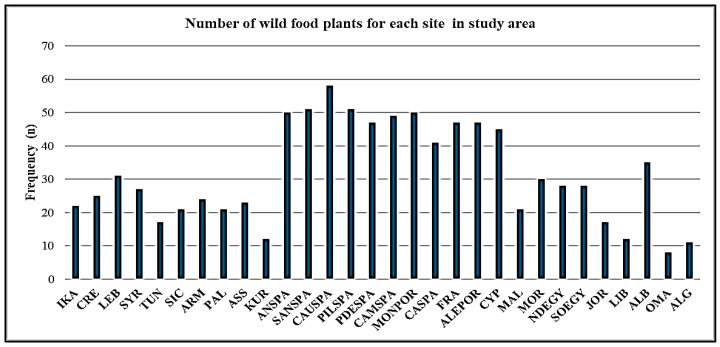
Frequency of wild food plant (genera) uses across study sites (Central Ikaria (IKA), Central Crete (CRE), Lebanon (LEB), Tartus (SYR), Sidi Bouzid (TUN), Sicily (SIC), Central Armenia (ARM), Northern West Bank (PAL), Syrian–Turkish borderland (ASS), Kurdistan region (KUR), Andalusia (ANSPA), Sanabria (SANSPA), Caurel (CAUSPA), Pilona (PILSPA), Picos de Europa (PDESPA), Campoo (CAMSPA), Montesinho (MONPOR), Catalonia (CASPA), Corsica (FRA), Alentejo (ALEPOR), Cyprus (CYP), Malta (MAL), Rif Mountains (MOR), Nile Delta (NDEGY), Siwa Oasis (SOEGY), Jordan Valley (JOR), Cyrenaica (LIB), Albanian South-East (ALB), Oman Mountains (OMA), and Western Algeria (ALG)).

The data reveal notable variations in the frequency of WFPs across the surveyed sites (Figure 3). The highest frequency was observed at CAUSPA, with 58 plants, accounting for 6.11% of the total dataset. Other significant contributors included San Spirito (SANSPA) with 51 plants (5.37%) and Andalusia (ANSPA) with 50 plants (5.27%). Sites like PILSPA, PDESPA, and CAMSPA also showed notable frequencies, each contributing around 51 to 58 plants (5.16% to 5.37%). Mid-range sites included LEB with 31 plants (3.27%), SYR with 27 plants (2.85%), TUN with 17 plants (1.79%), CRE with 25 plants (2.63%), IKA with 22 plants (2.32%), and SIC with 21 plants (2.21%).

In contrast, the lowest frequencies were recorded in Oman (OMA), with 8 plants (0.84%), and Algeria (ALG), with 11 plants (1.16%).

These findings underscore the diverse utilization of WFPs across the surveyed sites, reflecting the varied ecological contexts and cultural practices influencing their presence.

Table 2 highlights the cultural and culinary significance of various WFPs within the region, revealing several key findings. *Mentha* emerges as the most frequently used plant, appearing in 23 instances, valued for its versatility in teas, salads, sauces, and flavoring. *Sinapis* and *Smilax* also show high prevalence, used mainly in salads, as condiments, and for their young shoots in stews or pickling.

Leaves are the most commonly utilized plant part across the listed species, supporting their importance for both culinary applications and medicinal infusions. Other parts like roots and seeds also appear frequently, as seen with *Cichorium* roots used as a coffee substitute and *Foeniculum* seeds in teas and as a seasoning. Teas and infusions are especially popular, with plants such as *Mentha*, *Foeniculum*, *Origanum*, and *Thymus* reflecting a traditional emphasis on herbal beverages. Fresh dishes and salads are also common, with *Cichorium*, *Sinapis*, and *Eruca* frequently added as fresh greens, while *Thymus*, *Origanum*, and *Allium* are preferred for seasoning, indicating a tradition of enhancing flavors with local herbs. In addition, dual-use plants like *Malva*, *Urtica*, and *Artemisia* are used both for culinary and medicinal purposes, exemplifying the community’s extensive knowledge of the therapeutic properties of wild plants. The table also reflects the specialized knowledge required for safely consuming certain plants like *Bryonia* and *Cyclamen*, which have limited uses due to toxic properties. Overall, these results underscore the rich biodiversity of wild greens, their nutritional significance, and their integral role in local culinary traditions, highlighting the intricate relationship between the region’s flora and the cultural practices surrounding food.

### 3.2. Cluster Analysis of Wild Plant Distributions: Ecological and Geographical Insights Across Study Sites

**Figure 4:**
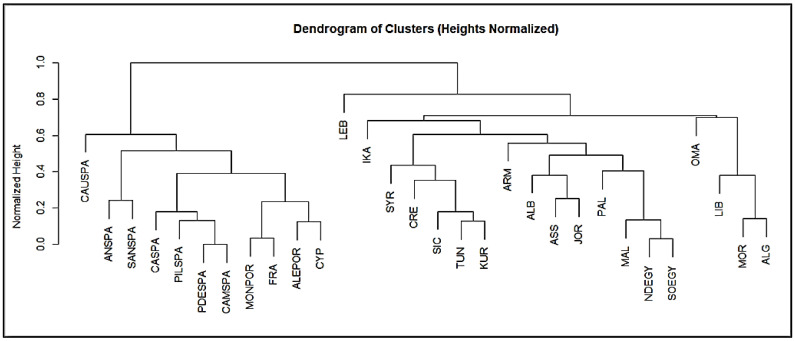
Dendrogram of wild plant distribution clusters among study sites (Central Ikaria (IKA), Central Crete (CRE), Lebanon (LEB), Tartus (SYR), Sidi Bouzid (TUN), Sicily (SIC), Central Armenia (ARM), Northern West Bank (PAL), Syrian–Turkish borderland (ASS), Kurdistan region (KUR), Andalusia (ANSPA), Sanabria (SANSPA), Caurel (CAUSPA), Pilona (PILSPA), Picos de Europa (PDESPA), Campoo (CAMSPA), Montesinho (MONPOR), Catalonia (CASPA), Corsica (FRA), Alentejo (ALEPOR), Cyprus (CYP), Malta (MAL), Rif Mountains (MOR), Nile Delta (NDEGY), Siwa Oasis (SOEGY), Jordan Valley (JOR), Cyrenaica (LIB), Albanian South-East (ALB), Oman Mountains (OMA), and Western Algeria (ALG)).

The cluster analysis of wild plant distributions across the 30 study sites represents hierarchical relationships and clustering of data points based on similarity or distance metrics, as illustrated in (Figure 4), revealing five main clusters based on their similarities.

The first cluster is the Western Mediterranean and Iberian Peninsula cluster: this cluster includes sites primarily from the Iberian Peninsula, such as Andalusia (ANSPA), Sanabria (SANSPA), Caurel (CAUSPA), Pilona (PILSPA), Picos de Europa (PDESPA), Campoo (CAMSPA), Montesinho (MONPOR), and Catalonia (CASPA), alongside Alentejo (ALEPOR) in Portugal and Corsica (FRA). These sites share a Mediterranean climate with considerable biodiversity adapted to the Iberian Peninsula’s unique topography. The prevalence of similar flora, such as species within the *Rosaceae* and *Lamiaceae* families, reflects a convergence in plant diversity shaped by similar environmental conditions, including moderate rainfall, warm temperatures, and high levels of sunlight. The shared cultural heritage across these sites has influenced the traditional use of wild plants for medicinal, culinary, and ritual purposes. Many of these areas have longstanding agricultural practices and traditional knowledge systems that emphasize the use of local plant species like *Malva* and *Thymus*. This is evident in the similar genera clustered within these regions, showcasing how geography and history intertwine to create shared ethnobotanical knowledge.

The second cluster includes the Eastern Mediterranean and Levantine: Lebanon (LEB), Syria (SYR), Cyprus (CYP), and the Kurdistan region (KUR) form a distinct cluster, often associated with the Levant and nearby regions. The Eastern Mediterranean has diverse microclimates that support a wide range of wild plant species, including endemic ones. Lebanon, for example, is known for its high plant biodiversity due to its varied topography and climate. Plants within families like *Lamiaceae* and *Apiaceae* are frequently used for traditional medicine and cooking, as they thrive in the Eastern Mediterranean’s relatively humid coastal zones and dry inland regions. This cluster shows that cultural factors are significant in shaping WFP usage. Countries in this region share a history of trade and cultural exchange that has contributed to common plant uses. For instance, herbs like *Origanum syriacum* are widely used in Lebanese, Palestinian, and Syrian cuisine, particularly in the preparation of the cultural dish of “Zaatar”. The deep-rooted cultural connections between these regions reinforce the consistent use of certain genera across countries.

The third cluster features North Africa: this group comprises sites in the North African region, including the Rif Mountains (MOR), the Nile Delta (NDEGY), Siwa Oasis (SOEGY), Cyrenaica (LIB), and Western Algeria (ALG). North Africa’s unique arid and semi-arid climates limit plant diversity compared to the more temperate zones in Europe. However, the presence of oases, such as Siwa, creates pockets of biodiversity that allow for the survival of species not typically found in desert regions. Plants such as *Ziziphus*, *Ficus*, and *Acacia* are commonly used in these areas, reflecting an adaptation to drought-prone environments. Traditional knowledge in North Africa emphasizes using drought-resistant plants for food, medicine, and ceremonial purposes. For example, *Ziziphus lotus* is utilized for its food and medicinal properties in Egypt and Libya. In addition, the influence of Amazigh and Arab cultures has led to distinct practices in herbal medicine, with plants like *Artemisia* spp. and *Peganum harmala* commonly used for health purposes and religious rituals.

The fourth is the island and mountainous regions cluster: Malta (MAL), Cyprus (CYP), Central Crete (CRE), and Central Ikaria (IKA) form a separate sub-cluster, possibly due to their island and mountainous characteristics. These island and isolated mountain regions are sometimes hotspots for endemic species that have evolved separately from mainland flora. Plants adapted to rocky soils, such as certain *Thymus* and *Sideritis* species, are prevalent in these areas. These regions are also subject to varying degrees of moisture from the surrounding seas, which supports a distinctive composition of WFPs. Islands like Crete and Ikaria have deep-rooted traditions in the use of wild plants, partly due to their geographical isolation, which fosters the preservation of old practices.

The last cluster is in the Arabian Peninsula: The Oman Mountains (OMA) form an isolated cluster, primarily due to their unique desert and mountainous environment in the Arabian Peninsula. The arid conditions and rugged topography of the Oman Mountains restrict the diversity of plant species, with many adapted to extreme temperatures and limited water availability. In Oman, traditional medicine and cultural practices still rely heavily on indigenous plants that are well adapted to the harsh local environment. The use of plants like *Boswellia sacra* for incense and medicine underscores the unique ethnobotanical traditions in the Arabian Peninsula, shaped by a combination of environmental constraints and cultural heritage.

However, the five clusters may be linked not only to plant ecology and distribution but also to cultural diffusion and customs. Considering, for example, that the wild greens portion of the recorded WFPs possibly originated in the Neolithic center of the Fertile Crescent and that during millennia, this system migrated westwards (Greece, then Italy, and later to the Iberian Penisula) and southwestwards (North Africa), the distinctive nature of the clusters linked to the Iberian, Levantine/Greek/South Italian and North African WFPs may reflect the different temporal steps that the Neolithic-centered foraging and consumption patterns went through in the journey from the East/Levant to the West Mediterranean.

**Figure 5:**
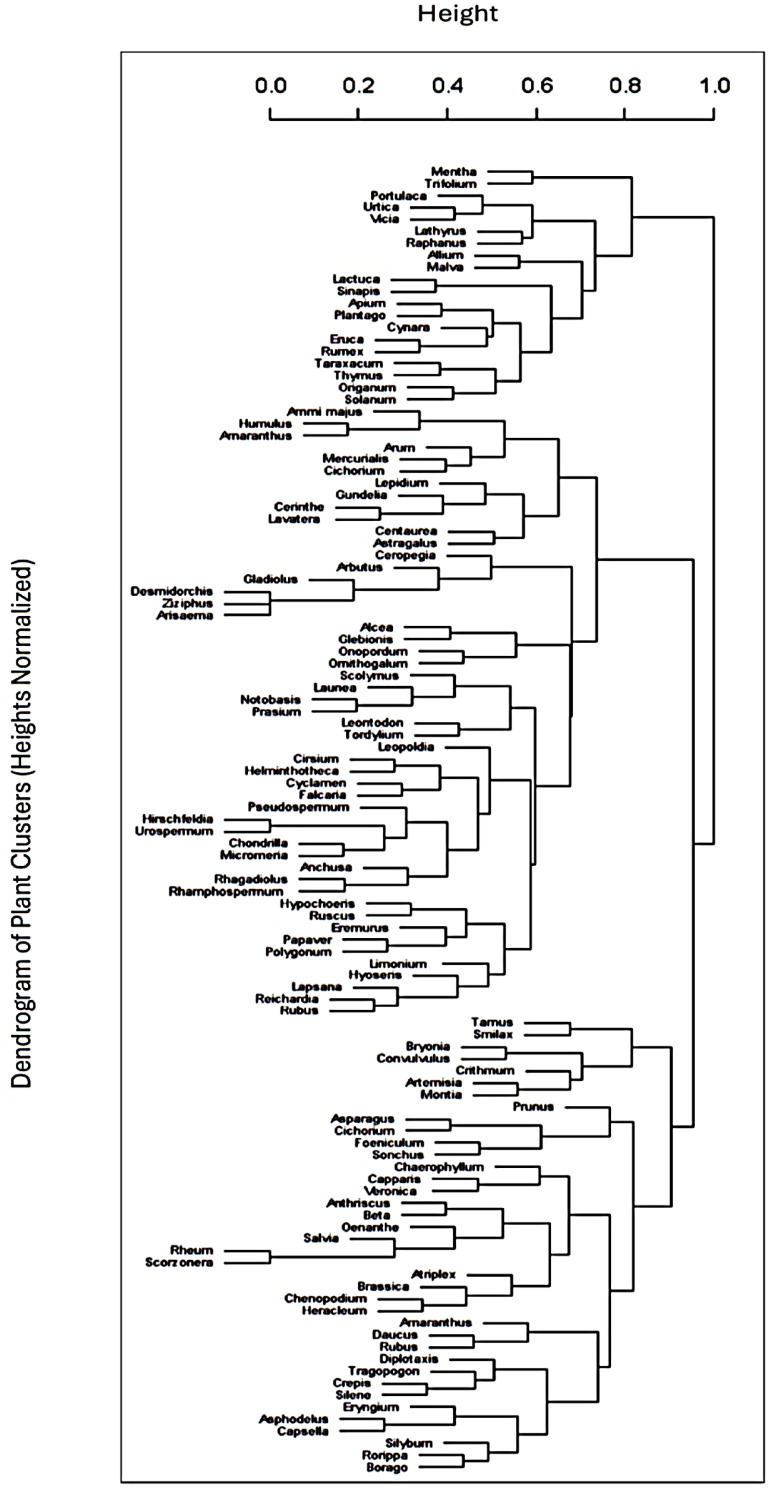
The dendrogram groups the WFP genera across the studied regions. This analysis aimed to capture the most frequently cited and widely used WFPs among different sites, providing insight into common taxa and usage patterns.

The analysis (Figure 5) reveals several clusters of genera that are commonly used across regions. For example, genera with high culinary value, such as *Malva*, *Chenopodium*, and *Pistacia*, form tightly knit clusters. These genera are recurrently found in various cultural contexts across the MENA regions, suggesting a shared ethnobotanical heritage. Some clusters highlight genera that are more specific to certain biogeographic zones. For instance, genera unique to the Eastern Mediterranean (e.g., *Origanum* in Lebanon and Syria) form distinct sub-clusters, reflecting the influence of local ecologies on WFP diversity. This clustering of WFP genera underscores the importance of both ecological availability and cultural familiarity in determining which WFPs are used in different regions. The repeated citation of specific genera also highlights their prominence in traditional diets and their potential resilience in diverse environmental contexts.

### 3.3. Phylogenetic Analysis and Ecological Clustering of Wild Food Plants

**Figure 6:**
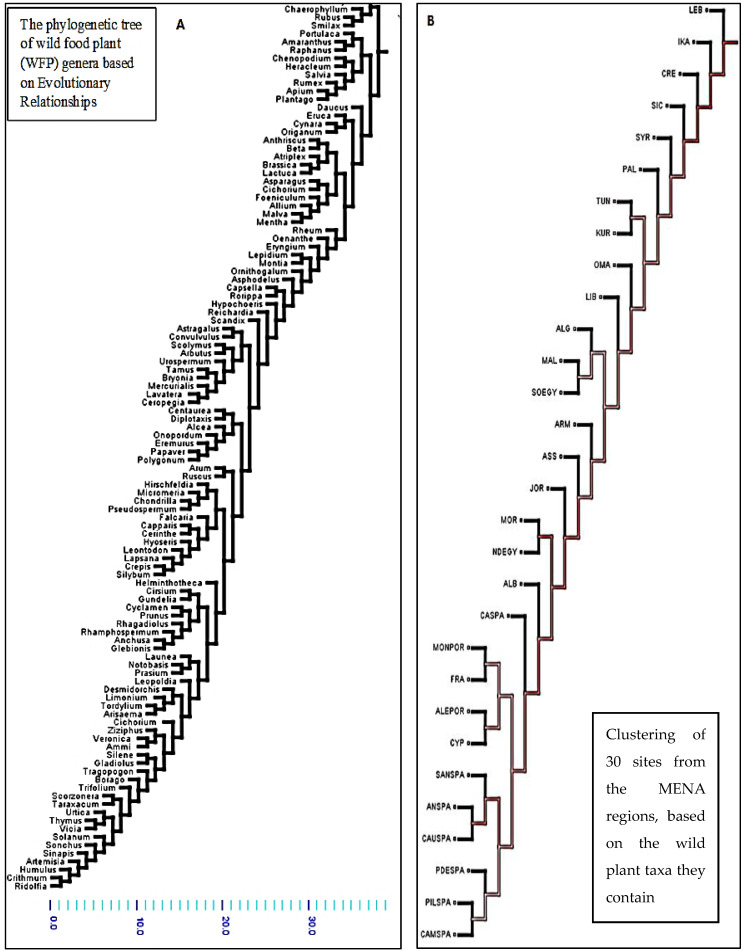
Phylogenetic relationships of WFPs and regional diversity across MENA sites: the phylogenetic clustering of WFPs and regional sites based on the wild plant diversity they harbor. (**A**) Panel represents the phylogenetic relationships among 111 wild plant species, where closely related taxa form distinct clusters, color-coded by divergence levels. (**B**) Panel illustrates the clustering of 30 sites from the MENA regions, based on the wild plant taxa they contain. (Central Ikaria (IKA), Central Crete (CRE), Lebanon (LEB), Tartus (SYR), Sidi Bouzid (TUN), Sicily (SIC), Central Armenia (ARM), Northern West Bank (PAL), Syrian–Turkish borderland (ASS), Kurdistan region (KUR), Andalusia (ANSPA), Sanabria (SANSPA), Caurel (CAUSPA), Pilona (PILSPA), Picos de Europa (PDESPA), Campoo (CAMSPA), Montesinho (MONPOR), Catalonia (CASPA), Corsica (FRA), Alentejo (ALEPOR), Cyprus (CYP), Malta (MAL), Rif Mountains (MOR), Nile Delta (NDEGY), Siwa Oasis (SOEGY), Jordan Valley (JOR), Cyrenaica (LIB), Albanian South-East (ALB), Oman Mountains (OMA), and Western Algeria (ALG)).

The phylogenetic tree (Figure 6A) of wild food plant (WFP) genera represents the evolutionary relationships among 111 wild food plant genera used across the 30 study sites in the MENA regions. By mapping these genera onto a phylogenetic tree, we can identify clusters that suggest both shared evolutionary history and functional adaptations, which in turn relate to their ecological distribution and cultural uses across the region based on common ancestry.

In the tree, some major plant families frequently used in traditional diets, such as *Lamiaceae*, *Rosaceae*, and *Fabaceae*, cluster together, illustrating their common ancestry and ecological versatility. These families are well adapted to the varied Mediterranean climates, from coastal to mountainous regions, and are often drought-tolerant and able to thrive in poor soils. Their adaptability has made them staples in local diets and traditional medicine, supporting uses ranging from culinary herbs to medicinal applications.

For instance, within the *Lamiaceae* family, genera like *Origanum*, *Salvia*, *Thymus*, and *Mentha* appear across multiple branches of the tree, showing evolutionary divergence within this family that likely correlates with adaptations to specific ecological niches.

Similarly, genera within *Rosaceae*, such as *Rubus* and *Rosa*, form another cluster, indicating their shared evolutionary history. These genera are found across varied environments from forests to arid zones and are commonly used in both culinary and medicinal applications. For example, wild berries are consumed as food, while roses are used in the preparation of herbal infusions and for their fragrance. This cluster highlights the resilience of *Rosaceae* plants and their adaptation to both Mediterranean and semi-arid environments.

Another notable cluster includes members of the Fabaceae family, such as Medicago and Trifolium, which are frequently used for foraging and as sources of protein in traditional diets. Fabaceae plants are nitrogen-fixing, enhancing soil fertility and thus playing an essential role in agroecological practices. This family’s evolutionary adaptations align well with traditional agricultural practices in the region, where they contribute to soil health and provide fodder.

Certain desert-adapted genera, such as *Ziziphus* (found in North Africa) and *Boswellia* (common in the Arabian Peninsula), form distinct branches that highlight their unique evolutionary paths, adapted to extreme arid conditions. These genera are particularly valued for their resilience and specific uses in harsh climates: *Ziziphus* fruits are nutritionally important, while *Boswellia* resin (frankincense) has deep cultural and medicinal significance, especially in Arabian cultures.

Overall, the phylogenetic tree shows how the evolutionary trajectories of different plant genera align with both ecological conditions and cultural practices in the MENA regions. This analysis reveals the intricate links between phylogeny, ecology, and ethnobotanical knowledge, emphasizing the deep-rooted cultural and biological connections to wild plants across diverse landscapes.

In (Figure 6B), the geographic distribution of WFPs (WFPs) across the MENA regions, as observed in the phylogenetic tree, reveals distinct clusters corresponding to natural biogeographic boundaries and historical human movements.

Lebanon, Ikaria, Crete, and Syria form a starting point for WFP diversity, exhibiting species that thrive in the arid and semi-arid climates typical of this area. The close grouping of these regions suggests shared environmental pressures and cultural practices that have influenced the use of similar plant species. The Kurdistan region (KURD), Syrian–Turkish borderlands (ASS), Malta (MAL), Cyprus (CYP), and Albania (ALB) show unique variability in WFPs. This diversity may reflect a mix of distinct cultural histories, microclimates, and unique flora adapted to local ecological conditions. For instance, Malta and Cyprus, as islands, have evolved flora that demonstrate both isolation effects and influences from nearby regions. The Kurdish region and Syrian–Turkish borderlands show connections to both Mediterranean and Middle Eastern plant traditions, reflecting their position at the cultural crossroads of these regions.

The connection observed between Malta (MAL) and Egypt (NDEGY, SOEGY) indicates a possible historical linkage in plant use and cultural exchange across the Mediterranean. This may stem from ancient trade routes that connected the North African coast with Mediterranean islands, allowing for the transfer of both plant species and ethnobotanical knowledge. The clustering of Spain and Portugal (e.g., Andalusia, Sanabria, Campoo, and Montesinho) reflects a unique Iberian flora shaped by the Western Mediterranean climate and centuries of agricultural tradition. This cluster includes plants adapted to both temperate and arid environments, pointing to a historical trajectory of WFP diversity that reflects the peninsula’s role as a hub of Mediterranean biodiversity. The distribution of WFPs here likely illustrates ancient agricultural pathways and connections across the region.

The distribution patterns seen in the tree suggest a historical trajectory of WFPs that mirrors human migration, trade, and cultural diffusion across the Mediterranean. From the Eastern Mediterranean (Lebanon, Ikaria, Crete, and Syria) westward to the Iberian Peninsula, these patterns reveal an ancient exchange network where both natural dispersal and human activity shaped the diversity and use of wild plants.

## 4. Discussion

### 4.1. Ecological, Cultural, and Climatic Influences on Wild Edible Plant Distribution

Our study identifies 111 WFPs across the MENA regions, revealing significant variations in their distribution and utilization. This discrepancy can be attributed to several factors, including local climate conditions, cultural practices, and historical reliance on foraging [69,70]. Areas with rich culinary traditions tend to integrate WFPs more extensively into their diets. In North Africa, while the region collectively accounts for 14% of citations, individual countries may show differences due to varying agricultural practices, dietary habits, and environmental conditions. We emphasize that sites with diverse habitats tend to support a greater variety of WFPs [14,17]. For instance, in regions with a combination of arid climates and coastal microclimates, such as parts of North Africa, we observe the prevalence of drought-resistant species like *Hordeum vulgare* and *Cichorium intybus*, which are well suited to these specific conditions. Conversely, in more temperate zones, such as parts of the Levant, the richness of plant species increases with the availability of water, leading to a broader variety of edible plants.

Similarly, Western Asia’s contribution of 12% reflects a blend of cultural significance and local availability of wild edibles in countries like Lebanon and Syria. However, certain communities may prioritize cultivated crops over foraged plants, leading to lower representation even within the same region [22].

Significant variations in the frequency of WFPs among different sites were observed. These discrepancies can be attributed to several factors: ecological context, as sites with diverse habitats support a greater variety of WFPs [71]; cultural practices, where regions with rich culinary traditions and a history of foraging demonstrate a stronger reliance on wild edibles [29,72]; and climate influence, where variations in microclimates within regions may facilitate plant diversity [23,34]. Additionally, human factors such as urbanization and agricultural practices can impact the availability of wild food plants, potentially leading to declines in foraging knowledge and, consequently, lower diversity, as observed in sites like Oman [28,70].

The cluster analysis reveals that the distribution of wild plant species across the MENA regions is shaped by both ecological factors and cultural traditions [43,73]. Each cluster highlights how specific environmental conditions such as arid climates in North Africa and the unique microclimates of island and mountainous regions promote the prevalence of certain wild plant species adapted to local conditions [56,63,74].

Additionally, shared cultural histories, particularly in the Western and Eastern Mediterranean, contribute to common uses and knowledge systems for wild plants [20,21,33], as seen in the culinary, medicinal, and ritual roles of species like *Malva*, *Origanum*, and *Thymus*. These findings underscore the importance of ecological adaptation and cultural continuity in shaping wild plant foraging and utilization [18,21,34]. Recognizing these interconnected influences not only enhances our understanding of ethnobotanical diversity but also highlights the potential for targeted conservation efforts that preserve both the ecological resilience and cultural significance of wild plants across these regions.

### 4.2. Phylogenetic Relationships and Ecological Clustering

The phylogenetic tree illustrates the evolutionary relationships among 111 wild food plant (WFP) genera used across 30 MENA study sites. Mapping these genera onto a phylogenetic tree highlights clusters that reveal shared evolutionary history, functional adaptations, and regional patterns in their ecological distribution and cultural usage. Several prominent plant families, *Lamiaceae*, *Rosaceae*, and *Fabaceae,* cluster together, suggesting their common ancestry and versatility across diverse Mediterranean environments [75]. These families, adapted to a range of climatic zones, have become integral to local diets and traditional medicine, with drought tolerance and resilience to poor soils aiding their widespread use. Within the *Lamiaceae* family, genera such as *Origanum*, *Salvia*, *Thymus*, and *Mentha* appear across multiple branches, suggesting evolutionary divergence that aligns with ecological niche adaptations across the Mediterranean [76,77]. Similarly, *Rosaceae* genera like *Rubus* and *Rosa* form a cluster that reflects their shared evolutionary history and ecological resilience, from forested to arid zones. These plants are culturally significant, with applications ranging from culinary uses (e.g., berries) to medicinal and aromatic uses (e.g., roses). The *Fabaceae* family, including genera such as *Medicago* and *Trifolium*, also forms a distinct cluster, showcasing its nitrogen-fixing ability and importance in agroecological practices, providing fodder and contributing to soil health. Additionally, desert-adapted genera like *Ziziphus* (North Africa) and *Boswellia* (Arabian Peninsula) form unique branches, illustrating their adaptations to arid conditions and cultural significance in local diets and traditional medicine. However, isolation can be attributed to geographical barriers or localized climatic conditions that encourage divergent evolutionary strategies [3,71].

While the phylogenetic analysis illustrated many significant results, we need to focus on the two core clusters of WFPs (WFP). These two cores appear to reflect distinct patterns of WFP diversity and usage across the MENA regions. The first core, centered around Lebanon and the Eastern Mediterranean, seems largely shaped by cultural diffusion, particularly the spread of agricultural practices and the Mediterranean diet from the west to the east. The second core, concentrated in the Western Mediterranean, highlights the role of biogeography and ecology in influencing the availability and types of WFPs.

#### 4.2.1. Eastern Mediterranean

The Eastern Mediterranean core, emerging from Lebanon and surrounding areas, points to a cultural diffusion model where agricultural practices and dietary traditions likely spread from east to west [38,75].

This movement aligns with historical evidence of early agricultural centers in the Fertile Crescent, where knowledge of cultivation, domestication, and plant use would have been disseminated across neighboring regions [22,78,79]. Here, the Mediterranean diet, characterized by a heavy reliance on olive oil, grains, fruits, and wild greens, may have laid the foundation for the integration of specific WFPs that were adaptable to local ecological conditions but had cultural origins further west [72]. The adoption of WFPs in this area might reflect an adaptation to the unique ecological conditions of the Eastern Mediterranean, which range from coastal zones to arid inland areas, while still preserving the culinary influence of the Mediterranean diet.

This core group illustrates how WFP use in the Eastern Mediterranean is deeply rooted in cultural tradition, likely influenced by ancient trade routes and migratory patterns that promoted the exchange of both cultivated and wild plants [6,78]. Additionally, the widespread adoption of the Mediterranean diet likely facilitated the movement of dietary preferences and specific WFPs, contributing to a regional continuity in the types of plants collected and their uses [34,72]. This connection underscores the importance of cultural continuity and diffusion in shaping WFP diversity and dietary practices.

#### 4.2.2. Western Mediterranean and North Africa

The second core group, centered around the Western Mediterranean and extending into North Africa, appears to be more strongly influenced by biogeographic and ecological factors. In this region, distinct climatic zones from the arid North African coasts to the temperate Iberian Peninsula create unique ecological niches that support diverse WFPs [14,59,66]. This ecological variability has driven the evolution of a distinctive set of wild plants suited to the region’s Mediterranean climate, including drought-resistant species and plants adapted to nutrient-poor soils.

Here, the collection and use of WFPs are likely shaped by local ecological constraints and opportunities rather than cultural diffusion alone [37,71,78]. Biogeography plays a central role, as the Western Mediterranean’s unique flora has developed in response to regional climate and topography. This ecological adaptation is seen in the diversity of plants used for foraging, which are often species well suited to arid and semi-arid conditions, reflecting the practical need for resilience in resource-scarce environments [23,70,72].

Furthermore, historical isolation due to geographic barriers such as mountains and seas has led to localized traditions of WFP use, with communities relying on plants native to their specific habitats. For instance, North African foragers may have developed a unique repertoire of drought-tolerant WFPs, such as edible cacti and hardy legumes, which differ from those found in wetter, northern parts of the Mediterranean. These patterns suggest that the Western Mediterranean core group is shaped not only by dietary tradition but by ecological necessity, with plant use practices being an adaptation to the region’s environmental conditions, and that was confirmed by many previous studies [25,37,65,71,76].

While these two core groups highlight different primary influences on cultural diffusion in the Eastern Mediterranean and biogeography in the Western Mediterranean, it is important to recognize the dynamic interplay between these factors [78]. The Mediterranean basin has long been a crossroads of trade, migration, and cultural exchange, which means that even as ecological conditions dictate the availability of certain WFPs, cultural practices, and knowledge transfer have also shaped plant use patterns [59,77].

For example, trade routes and migration paths likely allowed for the movement of both cultivated and wild plants across regions, leading to instances where culturally significant WFPs adapted to new ecological settings. This interplay suggests that while ecological constraints may determine which plants thrive in a given region, cultural preferences and dietary traditions help select which of these available plants are integrated into local diets and foraging practices.

### 4.3. Implications of Phylogenetic Relationships in Wild Plant Distribution

The findings of this study reveal significant implications regarding the distribution and conservation of wild plant diversity across various biogeographical zones. First, the close relationships observed between geographically distant regions suggest that cultural ties to post-Neolithic foraging (weeds) and biogeographical zones, rather than mere physical distances, play a crucial role in determining wild plant use distribution.

The Mediterranean climate emerges as a vital influence, extending its reach across North Africa and into parts of Western Asia. This highlights the interconnectedness of plant communities within these climatic zones, challenging the assumption that geographic separation inherently leads to distinct floristic profiles.

Furthermore, the clustering of regions such as North Africa with Southern Europe may reflect historical human activities, including trade and agricultural exchanges, which likely facilitated the dispersal of plant species across these areas. These cultural and historical connections underscore the dynamic relationship between humans and ecosystems over time.

Additionally, understanding the phylogenetic relationships among these regions has critical implications for conservation efforts. The conservation of certain wild plant species in one region could positively impact related species in other areas, especially in light of climate change, which threatens to disrupt the distribution patterns of Mediterranean and semi-arid ecosystems. By recognizing the interrelatedness of plant taxa across regions, conservation strategies can be developed that prioritize preserving genetic diversity and ecosystem resilience.

Recent developments in food security, biodiversity conservation, and sustainable agricultural practices have highlighted the critical role of wild food plants (WFPs) in enhancing ecosystem resilience and ensuring food security in the face of climate change [37,71]. As our study demonstrates, the diverse use of WFPs across the MENA region is not only a reflection of cultural heritage but also a potential strategy for adapting to global food system challenges [37]. WFPs are often resilient to climate stressors, including droughts and changing temperature patterns, making them valuable resources for sustainable agricultural practices. Integrating WFPs into modern farming systems can enhance biodiversity conservation by supporting diverse agroecosystems and promoting the use of native plants, which help preserve soil health and improve water retention [9,15]. Furthermore, the resurgence of interest in WFPs can help mitigate the loss of traditional agricultural knowledge and contribute to food sovereignty, particularly in regions heavily impacted by climate change and industrial agriculture [18]. By strengthening connections between local knowledge and scientific research on agroecology, we can develop more sustainable food systems that are both ecologically sound and culturally relevant, ensuring the long-term security of food sources across the MENA region and beyond.

On the other hand, some inevitable limitations may have had a considerable effect on our analysis, i.e., the heterogeneity of the focuses of the considered ethnobotanical works (in a few cases including all WFPs, in others only wild vegetables), the different nature of the field studies (some were conducted in very small areas and others in vast regions) and of the adopted field methodologies; moreover, some of the considered works for this analysis were conducted by well-trained ethnobotanists, others by agricultural and biological scholars.

## 5. Conclusions

This study highlights the intricate relationships between the phylogenetic history, ecological distribution, and cultural uses of wild food plants (WFPs) in the MENA regions. Through examining the evolutionary relationships among key genera, such as those in the *Lamiaceae*, *Rosaceae*, and *Fabaceae* families, we have identified significant clusters that demonstrate the resilience and ecological versatility of these plants in traditional diets. These families, adapted to the diverse climates of the region, have become deeply embedded in local cultures, with uses ranging from culinary to medicinal and agroecological applications. The geographical patterns observed in this study also reveal ancient cultural diffusion pathways, as seen in connections between regions such as Malta and Egypt, which likely facilitated the exchange of both plant species and ethnobotanical knowledge across the Mediterranean.

Our findings underscore the nutritional and culinary potential of WFPs and their role in diversifying modern food systems, offering a sustainable alternative to contemporary diets. Additionally, WFPs provide vital links between biodiversity conservation and the preservation of traditional ecological knowledge, highlighting their importance in maintaining cultural heritage. As global challenges such as climate change amplify the need for sustainable food systems, understanding the evolutionary, ecological, and cultural significance of these plants can guide the development of policies and strategies aimed at conserving biodiversity, promoting resilient agricultural practices and integrating WFPs into modern diets.

In conclusion, this study advocates for a holistic approach to the inclusion of WFPs in sustainable development efforts. By bridging the phylogenetic, ecological, and cultural dimensions of these plants, we provide a comprehensive framework for appreciating their contributions to both food security and biodiversity conservation, emphasizing the need for their inclusion in future research and conservation strategies across the MENA regions.

## Figures and Tables

**Table 1:** Ethnobotanical data on wild food plants by site, ethnicity, and religion in the MENA regions.

Site Code	Site	Country	Main Ethnicity	Religion	Actual Spoken Languages	References
IKA	Central Ikaria	Greece	Greek	Christianity	Greek	[33]
CRE	Central Crete	Greece	Greek	Christianity	Greek	[34]
LEB	Lebanon	Lebanon	Lebanese	Islam, Christianity	Arabic	[35,36]
SYR	Tartus	Syria	Syrian	Islam, Christianity	Arabic	[37]
TUN	Sidi Bouzid	Tunisia	Tunisian	Islam	Arabic	[38]
SIC	Sicily	Italy	Italian	Christianity	Italian	[39]
ARM	Central Armenia	Armenia	Armenian	Christianity	Armenian	[40]
PAL	Northern West Bank	Palestine	Palestinian	Islam, Christianity	Arabic	[41]
ASS	Syrian–Turkish borderland	Turkey/Syria	Assyrian	Islam, Christianity	Assyrian, Arabic, Kurdish	[42,43]
KUR	Kurdistan region	Iraq	Kurdish	Islam	Kurdish	[43]
ANSPA	Andalusia	Spain	Spanish	Christianity	Spanish	[44,45,46]
SANSPA	Sanabria	Spain	Spanish	Christianity	Spanish	[46]
CAUSPA	Caurel	Spain	Spanish	Christianity	Spanish	[44,45,46]
PILSPA	Pilona	Spain	Spanish	Christianity	Spanish	[44,45,46]
PDESPA	Picos de Europa	Spain	Spanish	Christianity	Spanish	[44,45,46,47]
CAMSPA	Campoo	Spain	Spanish	Christianity	Spanish	[44,45,46,47]
MONPOR	Montesinho	Portugal	Portuguese	Christianity	Portuguese	[46,48]
CASPA	Catalonia	Spain	Catalan	Christianity	Catalan, Spanish	[49,50]
FRA	Corsica	France	Corsican	Christianity	Corsican, French	[51]
ALEPOR	Alentejo	Portugal	Portuguese	Christianity	Portuguese	[46,52]
CYP	Turkish Cyprus	Cyprus	Turkish Cypriot	Islam	Turkish	[53]
MAL	Malta	Malta	Maltese	Christianity	Maltese	Wild food plants of Malta—index of plants with green flowers or flowerless https://www.maltawildplants.com/family_index.php, 12 November 2024 (maltawildplants.com)
MOR	Rif Mountains	Morocco	Berber	Islam	Arabic, Amazigh	[54,55,56]
NDEGY	Nile Delta	Egypt	Egyptian	Islam	Arabic	[57,58]
SOEGY	Siwa Oasis	Egypt	Siwi	Islam	Arabic, Siwi	[58,59,60]
JOR	Jordan Valley	Jordan	Jordanian	Islam	Arabic	[61,62]
LIB	Cyrenaica	Libya	Libyan	Islam	Arabic	[63]
ALB	Albanian South-East	Albania	Albanian	Islam, Christianity	Albanian	[32]
OMA	Oman Mountains	Oman	Omani	Islam	Arabic	[64,65,66]
ALG	Western Algeria	Algeria	Algerian	Islam	Arabic, Amazigh	[67,68]

**Table 2:** WFP genera were documented across a minimum of five sites in our study: diversity and culinary applications. (The percentage % in the context refers to the proportion of each botanical genus relative to the total number of WFPs documented across all study sites. It indicates how frequently a particular genus is represented in comparison to the overall dataset.

Wild Food Plant Genera	Frequency (*n*)	Percentage (%)	Used Parts	Local Culinary Uses
*Alcea*	6	0.53	Leaves, flowers, roots	Salads, medicinal teas, poultices
*Allium*	18	1.90	Leaves, bulbs	Soups, sauces, flavoring in numerous dishes
*Amaranthus*	13	1.37	Leaves, seeds	Soups, salads, stews, bread
*Anchusa*	5	0.42	Leaves, flowers	Stews, salads, garnishing
*Anthriscus*	14	1.48	Leaves, roots	Soups, stews, salads
*Apium*	12	1.26	Leaves, stems	Soups, stews, salads, as a garnish
*Artemisia*	9	0.95	Leaves, flowers	Teas, flavoring, medicinal uses
*Asparagus*	15	1.58	Young shoots	Steamed, grilled, soups, salads
*Asphodelus*	7	0.63	Roots, flowers	Cooked as vegetables, traditional food
*Astragalus*	7	0.74	Roots	Medicinal infusions, soups
*Atriplex*	12	1.26	Leaves, seeds	Salads, soups, stews
*Beta*	9	0.95	Leaves, roots	Salads, soups, stews, cooked greens
*Borago*	6	0.63	Leaves, flowers	Salads, soups, stews, decorative in culinary dishes
*Brassica*	15	1.58	Leaves, buds	Soups, stir-fries, salads
*Bryonia*	9	0.95	Shoots	Boiled
*Capparis*	8	0.84	Buds	Pickled as capers, seasoning
*Centaurea*	7	0.74	Flowers	Teas, salads, medicinal infusions
*Ceropegia*	6	0.63	Tubers	Steamed, traditional medicinal uses
*Chaerophyllum*	10	1.05	Leaves, roots	Soups, stews, salads, flavoring
*Chenopodium*	15	1.58	Leaves, seeds	Stews, soups, porridge, salads
*Cichorium*	20	2.11	Leaves, roots	Salads, cooked greens, coffee substitute (roots)
*Convulvulus*	10	1.05	Leaves	Salads, soups, medicinal purposes
*Crepis*	10	1.05	Leaves	Salads, soups, stews
*Crithmum*	11	1.16	Leaves, stems	Salads, pickled, as a garnish
*Cynara*	16	1.69	Flower buds, leaves	Steamed, baked, salads, appetizers
*Daucus*	12	1.26	Roots, leaves	Soups, salads, stews, flavoring
*Diplotaxis*	8	0.84	Leaves	Salads, stir-fries, as a garnish
*Eruca*	15	1.58	Leaves	Salads, pesto, sandwiches
*Eryngium*	7	0.74	Leaves, stems	Soups, stews, seasoning
*Foeniculum*	20	2.11	Leaves, seeds, stems	Teas, salads, soups, as seasoning
*Gundelia*	5	0.53	Stems, leaf midrib, flower heads	Stews, pickled, roasted in traditional dishes
*Heracleum*	13	1.37	Stems, leaves	Pickled stems, used as a spice
*Hyoseris*	5	0.53	Leaves	Salads, steamed as greens
*Lactuca*	17	1.79	Leaves	Salads, steamed greens
*Lapsana*	5	0.53	Leaves	Salads, soups
*Lathyrus*	18	1.90	Seeds, leaves	Soups, stews, traditional pasta dishes
*Leontodon*	7	0.74	Leaves, roots	Salads, teas, as greens
*Leopoldia*	5	0.53	Bulbs	Roasted, in stews, local delicacies
*Lepidium*	8	0.84	Leaves	Salads, seasoning, medicinal uses
*Limonium*	7	0.74	Leaves	Infusions
*Malva*	18	1.90	Leaves, flowers	Soups, salads, medicinal infusions
*Mentha*	23	2.42	Leaves	Teas, salads, sauces, and flavoring for dishes
*Montia*	11	1.16	Leaves, stems	Salads, soups
*Oenanthe*	10	1.05	Roots, leaves	Salads, soups, seasoning
*Onopordum*	6	0.63	Stems	Cooked as vegetable, in salads
*Origanum*	16	1.69	Leaves	Seasoning, salads, teas
*Ornithogalum*	8	0.84	Flowers, bulbs	Stews, soups, cooked as vegetables
*Papaver*	5	0.53	Seeds, Leaves	Boiled, bread, pastries, garnishing
*Plantago*	15	1.58	Leaves, seeds	Salads, soups, medicinal infusions
*Portulaca*	15	1.58	Leaves, stems	Salads, soups, stews
*Prunus*	17	1.79	Fruits, flowers	Jams, sauces, desserts, fermented drinks
*Raphanus*	19	2.00	Leaves, roots	Salads, pickles, seasoning with leaves
*Rheum*	11	1.16	Stems	Desserts, jams, sauces
*Rorippa*	8	0.84	Leaves, stems	Salads, as seasoning
*Rubus*	10	1.05	Fruits, leaves	Jams, desserts, teas
*Rumex*	16	1.69	Leaves	Soups, salads, stews, herbal teas
*Salvia*	13	1.37	Leaves	Teas, flavoring in dishes, medicinal use
*Scorzonera*	11	1.16	Roots	Steamed, soups, medicinal purposes
*Silene*	7	0.74	Leaves, stems	Soups, salads, stews
*Silybum*	6	0.63	Leaves, leaf midrib, stems	Steamed, soups, medicinal uses
*Sinapis*	21	2.21	Leaves, seeds	Salads, condiments, seed use in mustard preparation
*Smilax*	21	2.21	Young shoots, roots	Stews, soups, pickled shoots
*Solanum*	17	1.79	Berries, leaves	Fruits eaten raw or cooked, leaves in some cuisines
*Sonchus*	14	1.48	Leaves	Soups, salads, stews
*Tamus*	13	1.37	Shoots	Steamed, stews, soups after proper preparation
*Taraxacum*	17	1.79	Leaves, roots, flowers	Salads, teas, coffee substitute (roots)
*Thymus*	16	1.69	Leaves	Seasoning, teas, herbal infusions
*Tordylium*	6	0.63	Leaves, seeds	Seasoning, salads, soups
*Tragopogon*	10	1.05	Roots, leaves	Soups, stews, medicinal use
*Trifolium*	16	1.69	Leaves, flowers	Soups, teas, in salads
*Urtica*	17	1.79	Leaves	Soups, stews, herbal infusions after blanching
*Veronica*	6	0.63	Leaves	Soups, salads, herbal infusions
*Vicia*	18	1.90	Pods, seeds	Soups, stews, salads

## Data Availability

The original contributions presented in this study are included in the article. Further inquiries can be directed to the corresponding authors.
